# Cutaneous Diseases

**Published:** 1831-04-01

**Authors:** 


### Portfolio; or, Analecta Minora.

### I. Cutaneous Diseases.

In a clinical lecture, Dr. Elliotson threw out some important observations on the antiphlogistic treatment of diseases of the skin. A girl was admitted with impetigo of her arms and various parts of the body. She had head-ache, drowsiness, giddiness, and full pulse. She was put on very low diet and bled from time to time (the blood being generally inflamed)—while mercury was moderately exhibited. By this treatment, and this alone, the patient was soon cured of her impetigo, without any of the quackeries of local applications. In another case, where the eruption might be termed eczema, the same treatment was adopted, and the same results ensued. There is no doubt that the majority of cutaneous diseases are more than skin-deep, and would oftener give way to proper medicines internally exhibited, more especially with low diet, than to the farrago of external remedies.

